# A multicentric retrospective pilot study on the clinical, nutritional, economic and organizational impact of an integrated protocol for dysphagia management within nursing homes in Italy

**DOI:** 10.3389/fnut.2026.1882262

**Published:** 2026-07-15

**Authors:** Antonio Sebastiano, Chiara Mazzetti, Umberto Restelli, Sofia Silvola, Roberto Pigni, Carlo Pedrolli, Samir Giuseppe Sukkar

**Affiliations:** 1 Observatory on Nursing Homes, LIUC Cattaneo University, Castellanza, Italy; 2 Istituto La Provvidenza. Busto Arsizio, Italy; 3 Observatory on Value-Based Healthcare, LIUC Cattaneo University, Castellanza, Italy; 4 Nutrition Department, Santa Chiara Hospital, Azienda Provinciale per i Servizi Sanitari (APSS), Trento, Italy; 5 IRCCS Ospedale Policlinico San Martino, Genoa, Italy; 6 Dietetics and Clinical Nutrition Unit, University of Genoa, Genoa, Italy

**Keywords:** aspiration pneumonia, bioelectrical impedance analysis, body composition, health economics, integrated nutritional protocol, malnutrition risk, nursing home residents, oropharyngeal dysphagia

## Abstract

**Introduction:**

Dysphagia is a critical challenge in nursing homes, associated with malnutrition, aspiration pneumonia, and increased mortality. This study evaluates the impact of an integrated protocol combining instant homogenized meals, nutritional monitoring, and continuous staff training across five Italian nursing homes.

**Material and methods:**

A multicentric retrospective observational pilot study was conducted on 99 frail residents. The cohort was characterized by high functional dependence (88% total dependence) and severe nutritional vulnerability (99% malnutrited or at risk). Clinical evolution was compared between the 120 days preceding (pre-implementation) and the 120 days following the adoption of the protocol (post-implementation). Parameters analyzed included anthropometric data, bioelectrical impedance analysis (BIA), biochemical tests, adverse events (pneumonia, pressure ulcers), and economic/organizational impact in the post-implementation period (measured in two nursing homes).

**Results:**

In the post-implementation phase, an attenuation of weight decline was observed compared to the preceding period. BIA parameters showed favorable changes: phase angle increased significantly (*p* < 0.001), intracellular water rose while extracellular water decreased, and the ECM/BCM ratio improved, suggesting a more favorable body-composition and hydration pattern, although these findings should be interpreted cautiously given the possible influence of fluid shifts. Biochemically, increases in transferrin (*p* < 0.01), total protein, and albumin were recorded, supporting a coherent pattern of improved protein nutritional status. Clinically relevant adverse events decreased: aspiration pneumonia dropped from 8.2% to 5.1% (a 38.7% reduction), and pressure ulcers decreased from 24.7% to 19.2% (a 22.3% reduction). Economically, the cost per dietary day reduced by 17.5% (-€1.62), driven by a reduction in meal preparation time from 11 min 22 s to 3 min 21 s per guest. Qualitative analysis highlighted standardized consistencies, improved safety, and increased staff confidence. Likely, the integrated protocol was associated with a possible attenuation of nutritional decline in an extremely frail population, with coherent improvements in selected BIA-derived and biochemical markers and fewer recorded clinical complications.

**Discussion:**

Process standardization may enhance resident safety and operational efficiency, although these exploratory findings require confirmation in controlled prospective studies. These findings support managing dysphagia as a multidimensional clinical and organizational pathway rather than a simple texture modification strategy.

## Background

Dysphagia .is a symptom characterized by difficulty in forming or safely transporting the alimentary bolus from the mouth to the stomach, and it may result from oropharyngeal or oesophageal disorders. It is particularly frequent in older adults, in individuals with neurological and neurodegenerative conditions, and in subjects with head and neck diseases. In this population, dysphagia is clinically relevant not only because of its impact on swallowing safety, but also because it profoundly affects nutritional intake, hydration, mealtime experience, social participation, and overall quality of life. Malnutrition, dehydration, aspiration pneumonia, frailty, and increased mortality are among the most relevant adverse outcomes associated with dysphagia. Moreover, malnutrition is a recognized risk factor for pressure ulcers, further increasing the vulnerability of institutionalized older adults (1–6).

The burden of dysphagia is particularly relevant in nursing homes. Previous studies conducted in Italy and in the broader European long-term care setting have documented a substantial prevalence of dysphagia among residents, while systematic review evidence suggests that oropharyngeal dysphagia is especially common in nursing homes compared with other healthcare settings. This epidemiological picture is clinically important because nursing home residents often present with multimorbidity, cognitive impairment, severe functional dependence, and high nutritional vulnerability, all of which may amplify the adverse consequences of unsafe swallowing and poor oral intake (7–10).

The management of dysphagia in long-term care is therefore necessarily multidimensional. Current approaches typically include clinical assessment, texture-modified diets, thickened fluids, feeding assistance, postural strategies, nutritional monitoring, and staff training. However, the literature also shows that important gaps remain in real-world nursing home practice, particularly regarding staff upskilling, access to specialist support, and consistency standardization. In addition, non-standardized texture classification may expose residents to foods that are more difficult to swallow than intended, thereby potentially increasing choking risk and compromising intake. The implementation of internationally standardized frameworks such as IDDSI may therefore be particularly relevant in nursing homes, where operational variability can directly affect safety and nutritional adequacy (11–14).

The nutritional dimension of dysphagia management deserves particular attention. In long-term care, modified texture food has repeatedly been associated with a higher risk of malnutrition, and several studies have shown that residents receiving texture-modified meals may experience poorer nutritional intake and a high prevalence of micronutrient inadequacy. At the same time, the sensory quality, acceptability, and palatability of dysphagia diets can influence actual consumption and adherence. This means that meal safety alone is not sufficient: texture-modified nutrition should also be nutritionally dense, standardized, and acceptable to residents in order to be clinically effective (15, 16).

This rationale is supported by previous work from Sukkar et al. in a pilot study comparing instant homogenized meals with traditional pureed foods in patients with dysphagia, Sukkar et al. reported significantly better consumption and satisfaction in the group receiving instant homogenized meals, together with a lower frequency of dysphagia-related signs and symptoms during meals, despite the absence of major short-term differences in anthropometric and biochemical outcomes (17). These findings suggest that meal standardization and acceptability may represent clinically relevant targets even when short-term laboratory changes are modest. More recently, Pedrolli emphasized the need for clearer dysphagia-specific nutritional standards, particularly for breakfast, recommending explicit energy and protein targets, routine adoption of the IDDSI framework, and a multidisciplinary approach involving dietitians, speech-language pathologists, and nursing staff. Together, these contributions help frame dysphagia care not as a simple texture adjustment, but as an integrated nutritional and organizational pathway (18).

On this basis, the present study evaluated an integrated protocol for dysphagia management in Italian nursing homes, combining clinical assessment, instant homogenized meals, monitoring of intake, and continuous staff training. The primary aim was to quantify the clinical impact of this integrated approach compared with the previous management model. Secondary aims were to estimate the economic consequences from the nursing home perspective and to assess the organizational implications of protocol implementation.

## Materials and methods

A multicentric retrospective observational pilot study approved by the ethic committee CET 5 Lombardia on the 22*^nd^* April 2025 (study protocol DISF_RSA_2024, protocol number 53/25) was implemented including residents of the five participating Italian nursing homes: Istituto La Provvidenza ONLUS, Busto Arsizio, VA; Villaggio Amico Srl, Gerenzano, VA; Istituto Cav. Francesco Menotti ONLUS, Cadegliano-Viconago, VA; Fondazione Elisabetta Germani ONLUS, Cingia De’ Botti, CR; Fondazione Villa Sacro Cuore Coniugi Preyer ONLUS, Casalmorano, CR. The study was conducted in accordance with the ethical principles of the Declaration of Helsinki and applicable Italian regulations for observational research.

The study was based on retrospective data, and all information were anonymized prior to analysis and processed in aggregated form to ensure confidentiality. Moreover, participating facilities authorized the use of institutional data for research purposes, and informed consent for the use of clinical information had been previously obtained according to local institutional procedures.

The analytical design should therefore be interpreted as a retrospective single-group before-and-after comparison of the same cohort over two consecutive 120-day periods, without randomization and without a concurrent control group. Consequently, the study was conceived as an exploratory, hypothesis-generating pilot evaluation of feasibility and short-term associations, rather than as a confirmatory efficacy study.

Subjects were included if they were dysphagic, who had been fed according to a specific protocol for at least 120 days before the adoption of the integrated protocol for dysphagia management under assessment, and for whom a 120-day observation period after implementation of the integrated protocol was available; subjects who signed an informed consent to participate to the study. Exclusion criteria included terminal illness, oncological diseases, cirrhosis, severe cardiovascular diseases, and the use of enteral nutrition. All residents meeting the eligibility criteria and who had adopted the integrated protocol between 1 January 2024 and 15 August 2024 were included.

Dysphagia was identified within each nursing home according to routine clinical practice, mainly on the basis of clinical observation and local care documentation. Standardized information on the diagnostic procedure, the professional profile of the assessor, instrumental swallowing assessment (VFSS or FEES), validated bedside swallowing scales, dysphagia severity grading, and participant-level IDDSI consistency levels was not available in the retrospective records. This limits stratification by dysphagia severity and should be considered when interpreting aspiration-related outcomes.

Baseline information of residents were collected on the date in which the integrated protocol for dysphagia management was implemented, including demographic data, weight, height, BMI, bioelectrical impedance analysis (BIA) parameters, blood test, complete leukocyte differential count, biochemical parameters, nutritional risk assessment either through Mini Nutritional Assessment (MNA) (19) or Malnutrition Universal Screening Tool (MUST) (20), assessment of functional independence in activities of daily living (ADL) through Barthel Index (21), assessment of pressure ulcer risk (Braden Scale or Norton Scale) (22, 23), assessment of dementia severity through Clinical Dementia Rating (CDR) (24). Data were also collected using the values closest to 120 days before and after the baseline date.

The change between the start of the observation period (120 days before implementation of the integrated protocol for dysphagia management) and baseline was compared with the change between baseline and 120 days after implementation of the protocol. Furthermore, a sample of food diaries were analyzed to assess meal consumption.

The incidence of clinical events was retrieved from the clinical records recorded within each nursing home in the two period under assessment. The clinical events considered are pneumonia ab ingestis and pressure ulcers.

Economic data related to secondary objective were collected using an *ad hoc* extracontable survey instrument developed by the research team and validated in previous economic analyses. The tool requires the collection of the main cost drivers of catering services, including food ingredients, preparation processes, staff time, and operational resources used in meal production, together with information on dietary characteristics of residents and the organization of the food service. Economic data referred to the full year 2023 and to the first semester of 2024 were considered, representing the most recent period available at the time of analysis. Due to the structure of accounting systems adopted by the participating facilities, it was not always possible to isolate costs specifically attributable to meals prepared for residents with dysphagia prior to protocol implementation, therefore, baseline cost estimates for the traditional preparation model were calculated using data referring to the overall catering service. The differential economic impact related with the adoption of the integrated protocol for dysphagia management was estimated based on process analysis conducted withing two participating nursing homes, assessing the resources adopted in each micro phase of the meal preparation processes (including machine predisposition, meal preparation and machine washing).

Organizational implications were assessed through qualitative semi-structured interviews conducted within the five participating facilities (25). Interviewees (recruited on a voluntary basis) included a multidisciplinary group of professionals involved in dysphagia management, such as physicians (2), medical directors (4), nursing coordinators (3), kitchen managers and staff (4), personnel responsible for preparing dysphagia-specific meals (5), procurement managers (4), and, in one facility, the general director (1). Interviews (with a duration of 30/45 min) were conducted in person by two researchers with expertise in healthcare management and health economics using a predefined interview guide addressing workflow changes, staff workload, operational challenges, and perceived benefits associated with the newly adopted integrated protocol. Responses were documented through notes and independently reviewed by two researchers to identify recurring themes related to organizational impact. Discrepancies were resolved through discussion and consensus.

Quantitative data were analyzed using descriptive statistical methods such as frequencies, means, medians, interquartile ranges, standard deviations and confidence intervals (95%). Clinical indicators and economic variables were compared between the periods preceding and following the implementation of the integrated dysphagia management protocol. Differences within the two phases (baseline – 120 days pre-adoption; 120 days post-adoption - baseline) were evaluated using paired *t*-tests for normally distributed variables and Wilcoxon Signed-Rank Test for not normally distributed variables. Furthermore, effect size was calculated using Cohen’s *d* test for normally distributed variables; and derived from Wilcoxon Signed-Rank Test for not normally distributed variables. No correction methods were applied given the exploratory nature of the study. Where appropriate, absolute and relative differences were calculated to describe variations associated with the introduction of the integrated protocol.

Because of the retrospective pilot design and the limited sample size, no multivariable adjustment, mixed-effects modeling, facility-level adjustment, or imputation of missing values was performed. Analyses were based on available paired observations for each outcome, and the number of evaluable subjects is reported for each variable. The results should therefore be interpreted as exploratory associations that may be affected by missing data, residual confounding, within-facility practices, seasonality, survivor bias, and other unmeasured co-interventions.

Qualitative data from the interviews were analyzed through thematic synthesis in order to identify recurring organizational themes related to workflow changes, staff workload, and operational implications.

## Results

Ninety-nine subjects were included in the analysis. Baseline characteristics are reported in Table 1.

**TABLE 1:** Baseline patients characteristics.

Variable	Total sample	Value
**Age (years)**		
Mean (±standard deviation)	99 subjects	85.38 ( ± 9.44)
Median	99 subjects	87.0
Sex – number (%)	99 subjects	
Females	–	77 (77.8%)
Males	–	22 (22.2%)
Weight (Kg) – mean (±SD)	99 subjects	51.63 (±11.28)
BMI – mean (±SD)	99 subjects	20.53 (±4.49)
MNA scale – number (%)	88 subjects	
Protein-calorie malnutrition: <17	–	31 (35%)
At risk for malnutrition: 17–23.5	–	56 (64%)
Good nutritional status: 24–30	–	1 (1%)
MUST Scale – number (%)	11 subjects	
Low malnutrition risk: 0	–	2 (18%)
Medium malnutrition risk: 1	–	2 (18%)
High malnutrition risk: ≥2	–	7 (64%)
Modified BARTHEL index – number (%)	99 subjects	–
Total dependence: 0–24	–	87 (88%)
Severe dependence: 25–49	–	12 (12%)
Moderate dependence: 50–74	–	0 (0%)
Mild dependence: 75–90	–	0 (0%)
Minimal dependence: 91–99	–	0 (0%)
BRADEN scale – number (%)	98 subjects	–
Very high risk: ≤6	–	1 (1%)
High and moderate risk: 7–18	–	86 (88%)
Low risk: 19–23	–	11 (11%)
NORTON scale – number (%)	15 subjects	–
High risk: ≤11	–	13 (87%)
Moderate risk: 12–14	–	2 (13%)
Low risk: ≥ 15	–	0 (0%)
CDR scale – number (%)	98 subjects	–
No cognitive impairment: 0	–	0 (0%)
Questionable cognitive impairment: 0.5	–	0 (0%)
Mild dementia: 1	–	4 (4%)
Moderate dementia: 2	–	6 (6%)
Severe dementia: 3	–	41 (42%)
Very severe dementia: 4	–	47 (48%)
Terminal dementia: 5	–	0 (0%)
**Bioimpedance parameters**		
PA (°)–mean (±SD)	98 subjects	3.16 (±0.70)
TBW (%)–mean (±SD)	98 subjects	60.47 (±8.29)
ICW (%)–mean (±SD)	98 subjects	35.54 (±6.08)
ECW (%)–mean (±SD)	98 subjects	64.04 (±7.50)
SMM (%)–mean (±SD)	98 subjects	5.11 (±5.11)
FFM (%)–mean (±SD)	98 subjects	74.18 (±13.16)
BCM (%)–mean (±SD)	98 subjects	7.60 (±4.68)
ECM (%)–mean (±SD)	98 subjects	66.58 (±13.72)
FM (%)–mean (±SD)	98 subjects	23.73 (±10.34)
ECM/BCM (%)–mean ( ± SD)	84 subjects	17.21 (±12.26)
Sodium-potassium exchanger (Na^+^/K+)–mean (±SD)	98 subjects	1.95 (±0.54)
BCMI (%)–mean (±SD)	98 subjects	4.77 (±2.86)
BMR (kcal)–mean (±SD)	98 subjects	1,133.48 (±114.16)
**Biochemical parameters**		
WBC (mil/mm^3^)	96 subjects	6.11 (±1.84)
RBC (mil/mm^4^)	96 subjects	4.04 (±0.54)
Hb (g/dl0	96 subjects	12.21 (±1.35)
HCT (%)	96 subjects	37.01 (±4.14)
PLT (N°/μl)	96 subjects	235.67 (±98.78)
Total protein (g/dl)	75 subjects	6.37 (±0.63)
MCV (Fl)	96 subjects	91.33 (±9.43)
MCH (Pg)	96 subjects	30.43 (±2.34)
MCHC (%)	96 subjects	33.04 (±1.10)
RDW (%)	96 subjects	14.42 (±1.14)
Azotemia (mg/dL)	82 subjects	51.26 (±29.73)
Creatinine	95 subjects	0.97 (±0.46)
Albumin	83 subjects	3.53 (±0.35)
CRP	89 subjects	2.77 (±8.61)
Transferrin	80 subjects	189.54 (±32.63)
Blood glucose	78 subjects	81.63 (±15.93)

BCM, body cell mass; BCMI, body cell mass index referred to height; BMI, body mass index; BMR, basal metabolic rate, CPR, C-reactive protein; ECM, extracellular mass; ECW, extracellular water; FFM, fat-free mass; FM, fat mass; Hb, hemoglobin; HCT, hematocrit; ICW, intracellular water; MCH, mean corpuscular hemoglobin; MCHC, mean corpuscular hemoglobin concentration; MCV, mean corpuscular volume; PA, phase angle; PLT, platelets; RBC, red blood cell; RDW, red cell distribution width; SD, standard deviation; SMM, skeletal muscle mass; TBW, total body water; WBC, white blood cell.

Subjects included in the analysis were mainly females (77.8%) with a mean age of 85.38 (±9.44) years. Malnutrition risk was mainly assessed through MNA, and the risk to develop pressure ulcers was mainly assessed through BRADEN Score.

The baseline profile of the study population depicts an extremely frail and nutritionally vulnerable cohort. According to the Mini Nutritional Assessment, 35% of residents were already in the range of overt protein-calorie malnutrition and 64% were at risk of malnutrition, while only 1% showed a normal nutritional status. This nutritional impairment was coherent with body composition findings assessed by bioelectrical impedance analysis. In particular, the mean phase angle was markedly reduced (3.16°), suggesting severely compromised cellular integrity, and the mean BCMI value was also consistent with profound nutritional depletion. Biochemical findings supported this interpretation, showing a pattern compatible with protein malnutrition on a background of chronic inflammatory burden, as reflected by low transferrin levels (189.54 ± 32.63 mg/dL), borderline-low albumin, and elevated CRP variability. The clinical severity of the cohort was further confirmed by functional data: 88% of residents were totally dependent according to the modified Barthel Index and the remaining 12% showed severe dependence, with no residents in the moderate, mild, or minimal dependence categories. Finally, the risk of pressure ulcers was particularly high, with 88% of residents classified at moderate-to-high risk and 1% at very high risk.

### Clinical assessment

**FIGURE 1:**
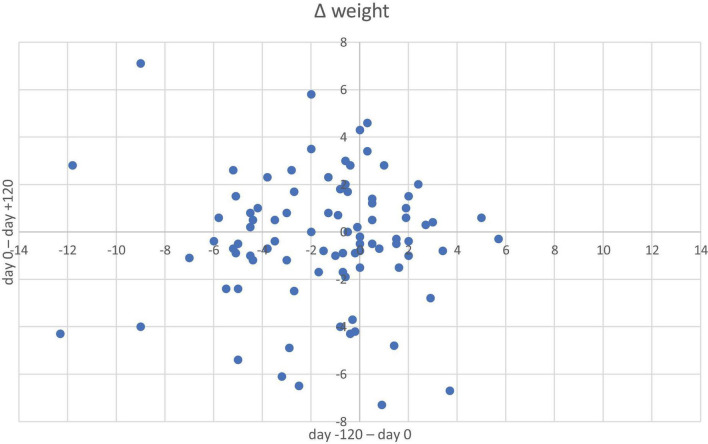
Subjects distribution considering weight change in the pre and post implementation phase.

The mean variations between day −120 and baseline (pre implementation phase) and between baseline and day +120 (post implementation phase) of parameters related to weight, BMI, bioimpedance and biochemical tests are reported in Table 2 and in Figure 1.

**TABLE 2:** Parameters variations related to weight, BMI, bioimpedance, and biochemical tests.

Variable	Parameter variation		
	Period variation	Effect size^	*P*-value^#^
Weight (Kg)			
Pre (day −120; 0): median (IQR) (*N*: 94)	−0.75 (4.00)	−0.419	0.0001***
Post (day 0; +120): median (IQR) (*N*: 96)	−0.45 (2.95)	−0.172	0.0949
BMI			
Pre (day −120; 0): median (IQR) (*N*: 94)	−0.31 (1.68)	−0.430	0.0000***
Post (day 0; +120): median (IQR) (*N*: 96)	−0.17 (1.19)	−0.166	0.1071*
Bioimpedance parameters – post implementation phase			
PA (°) - mean (±SD) [CI 95%] (*N*: 94)	+0.30 (±0.54) [0.194; 0.414]	0.566	0.0000***
TBW (%) - median (IQR) (*N*: 96)	−1.30 (5.90)	−0.220	0.0331*
ICW (%) - mean (±SD) [CI 95%] (*N*: 95)	+2.58 (±4.79) [1.609; 3.560]	0.540	0.0000***
ECW (%) - mean (±SD) [CI 95%] (*N*: 96)	−2.60 (±4.71) [−3.557; −1.647]	−0.552	0.0000***
SMM (%) - median (IQR) (*N*: 96)	−0.90 (2.35)	−0.410	0.0001***
FFM (%) - median (IQR) (*N*: 96)	+0.85 (6.55)	0.223	0.0301*
BCM (%) - median (IQR) (*N*: 96)	+0.40 (1.65)	0.289	0.0061**
ECM (%) - median (IQR) (*N*: 96)	+1.10 (6.55)	0.216	0.0363*
FM (%) - median (IQR) (*N*: 96)	−0.85 (6.55)	−0.240	0.0204*
ECM/BCM (%) - median (IQR) (*N*: 82)	−17.82 (24.44)	−0.777	0.0000***
Sodium-potassium exchanger [Na^+^/K^+^] - median (IQR) (*N*: 96)	−0.14 (0.40)	−0.534	0.0000***
BCMI (%) - median (IQR) (*N*: 96)	+0.26 (1.06)	0.292	0.0056**
BMR (kcal) - mean (±SD) (CI 95%) (*N*: 96)	+27.10 (±76.14) [11.670; 42.524]	0.356	0.0007***
Biochemical parameters			
Pre (day −120; 0): WBC (mil/mm^3^) - median (IQR) (*N*: 65)	−0.20 (1.43)	−0.153	0.2217
Post (day 0; +120): WBC (mil/mm^3^) - median (IQR) (*N*: 74)	+0.09 (1.38)	0.156	0.1824
Pre (day −120; 0): RBC (mil/mm^4^) - mean (±SD) [CI 95%] (*N*: 65)	+0.03 ( ± 0.31) [-0.051; 0.103]	0.085	0.4978
Post (day 0; +120): RBC (mil/mm^4^) - mean (±SD) [CI 95%] (*N*: 74)	+0.09 ( ± 0.33) [0.012; 0.167]	0.268	0.0240*
Pre (day −120; 0): Hb (g/dl) - mean ( ± SD) [CI 95%] (*N*: 65)	−0.05 ( ± 0.81) [−0.254; 0.147]	−0.067	0.5934
Post (day 0; +120): Hb (g/dl) - mean ( ± SD) [CI 95%] (*N*: 74)	+0.02 ( ± 0.98) [−0.203; 0.252]	0.025	0.8316
Pre (day −120; 0): HCT (%) - mean (±SD) [CI 95%] (*N*: 65)	+0.18 (±2.50) [−0.445; 0.795]	0.070	0.5740
Post (day 0; +120): HCT (%) - mean (±SD) [CI 95%] (N: 74)	+0.40 (±2.92) [−0.281; 1.073]	0.136	0.2474
Pre (day −120; 0): PLT (N°/μl) - mean (±SD) [CI 95%] (N: 65)	−13.91 ( ± 54.21) [−27.340; -0.475]	−0.257	0.0427*
Post (day 0; +120): PLT (N°/μl) - median (IQR) (N: 74)	−1.50 (59.00)	−0.062	0.5956
Pre (day −120; 0): total protein (g/dl) - mean ( ± SD) [CI 95%] (N: 18)	−0.01 (±0.42) [−0.217; 0.197]	−0.024	0.9201
Post (day 0; +120): total protein (g/dl) - mean ( ± SD) [CI 95%] (*N*: 53)	+0.23 (±0.59) [0.063; 0.388]	0.382	0.0075**
Pre (day −120; 0): MCV (Fl) - median (IQR) (*N*: 65)	−0.30 (3.50)	−0.133	0.2957
Post (day 0; +120): MCV (Fl) - median (IQR) (*N*: 74)	−0.25 (4.50)	−0.220	0.0623
Pre (day −120; 0): MCH (Pg) - median (IQR) (*N*: 65)	−0.30 (0.90)	−0.278	0.0276*
Post (day 0; +120): MCH (Pg) - mean (±SD) [CI 95%] (*N*: 74)	−0.66 ( ± 1.48) [−1.004; −0.320]	−0.448	0.0002***
Pre (day −120; 0): MCHC (%) - median (IQR) (*N*: 65)	−0.20 (0.90)	−0.244	0.0550
Post (day 0; +120): MCHC (%) - mean (±SD) [CI 95%] (*N*: 74)	-0.32 ( ± 1.07) [-0.568; -0.073]	−0.300	0.0119*
Pre (day −120; 0): RDW (%) - median (IQR) (*N*: 64)	−0.10 (1.25)	−0.059	0.6468
Post (day 0; +120): RDW (%) - median (IQR) (*N*: 73)	+0.10 (1.30)	0.158	0.1906
Pre (day −120; 0): azotemia (mg/dL) - median (IQR) (*N*: 40)	+3.00 (17.00)	0.303	0.0654
Post (day 0; +120): azotemia (mg/dL) - median (IQR) (*N*: 59)	+5.00 (14.00)	0.391	0.0031**
Pre (day −120; 0): creatinine - median (IQR) (*N*: 60)	−0.01 (0.15)	0.021	0.8742
Post (day 0; +120): creatinine - median (IQR) (*N*: 73)	−0.04 (0.15)	−0.440	0.0003***
Pre (day −120; 0): albumin - mean (±SD) [CI 95%] (*N*: 32)	0.00 (±0.39) [−0.141; 0.141]	−0.000	1.0000
Post (day 0; +120): albumin - mean (±SD) [CI 95%] (*N*: 57)	+0.09 (±0.32) [0.005; 0.177]	0.281	0.0385*
Pre (day −120; 0): CRP - median (IQR) (*N*: 18)	−0.01 (2.56)	0.127	0.6012
Post (day 0; +120): CRP - median (IQR) (*N*: 66)	+0.01 (0.82)	0.070	0.5805
Pre (day −120; 0): transferrin - median (IQR) (*N*: 26)	−2.50 (26.00)	−0.057	0.7699
Post (day 0; +120): transferrin - median (IQR) (*N*: 58)	+6.00 (29.00)	0.397	0.0029**
Pre (day −120; 0): blood glucose - mean (±SD) [CI 95%] (*N*: 27)	−1.19 ( ± 9.86) [-5.084; 2.714]	−0.120	0.5376
Post (day 0; +120): blood glucose - median (IQR) (*N*: 60)	+1.00 (14.00)	0.179	0.1812

^Effect size: Cohen’s *d* test used for normally distributed variables; effect size r derived from Wilcoxon Signed-Rank Test for not normally distributed variables. ^#^*P*-values were calculated using paired *t*-tests for normally distributed variables and Wilcoxon Signed-Rank Test for not normally distributed variables. BCM, body cell mass; BCMI, body cell mass index referred to height; BMI, body mass index; BMR, basal metabolic rate; CI, confidence interval; CPR, C-reactive protein; ECM, extracellular mass; ECW, extracellular water; FFM, fat-free mass; FM, fat mass; Hb, hemoglobin; HCT, hematocrit; ICW, intracellular water; IQR, interquartile range; MCH, mean corpuscular hemoglobin; MCHC, mean corpuscular hemoglobin concentration; MCV, mean corpuscular volume; PA, phase angle; PLT, platelets; RBC, red blood cell; RDW, red cell distribution width; SD, standard deviation; SMM, skeletal muscle mass; TBW, total body water; WBC, white blood cell.

*P-value < 0.05.

**P-value < 0.01.

***P-value < 0.001.

After 120 days from implementation of the integrated protocol for dysphagia management, an important change in the trajectory of nutritional decline was observed. Although body weight and BMI still decreased on average, the magnitude of decline was lower than in the pre-implementation period, suggesting clinical attenuation of ongoing nutritional deterioration. This stabilization was accompanied by a favorable shift in several bioimpedance-derived parameters. Most notably, phase angle increased significantly, intracellular water increased while extracellular water decreased, and the ECM/BCM ratio improved markedly, all of which are consistent with better cellular function and a more favorable hydration pattern. BCM and BCMI also showed modest positive changes, while FFM increased and FM decreased. Overall, these findings suggest an improvement in body composition quality and in the active cellular compartment, even in the absence of frank weight recovery.

Biochemical parameters were directionally consistent with this pattern. In the post-implementation phase, total protein, transferrin, red blood cell count, and albumin tended to improve, while CRP remained rather stable and creatinine decreased significantly. Taken together, these data suggest a likely improved nutritional intake and a more favorable metabolic profile. In particular, the increase in transferrin may reflect better protein nutritional status, whereas the lack of a parallel increase in creatinine despite the rise in blood urea nitrogen is compatible with the interpretation that higher azotemia may have been related, at least in part, to increased protein intake rather than to incipient renal impairment. This interpretation should nevertheless be considered cautiously in a frail elderly population.

The clinical assessment tools’ scores variation during the pre and post implementation phases are reported in Table 3.

**TABLE 3:** Subjects distribution among scores variations related to clinical assessment tools.

Scale	Subjects distribution	
	Pre implementation phase (day−120; 0)	Post implementation phase (day 0; +120)
MNA scale – number (%)		
Score improvement	6 (6.1%)	9 (9.1%)
Score worsening	18 (18.2%)	9 (9.1%)
Stable score	60 (60.6%)	64 (64.6%)
Not applicable	15 (15.2%)	17 (17.2%)
MUST scale – number (%)		
Score improvement	1 (1.0%)	2 (2.0%)
Score worsening	4 (4.0%)	0 (0.0%)
Stable score	6 (6.1%)	9 (9.1%)
Not applicable	88 (88.9%)	88 (88.9%)
BARTHEL Index – number (%)		
Score improvement	2 (2.0%)	0 (0.0%)
Score worsening	5 (5.1%)	4 (4.0%)
Stable score	91 (91.9%)	92 (92.9%)
Not applicable	1 (1.0%)	3 (3.0%)
BRADEN scale – number (%)		
Score improvement	2 (2.0%)	4 (4.0%)
Score worsening	6 (6.1%)	3 (3.0%)
Stable score	71 (71.7%)	88 (88.9%)
Not applicable	20 (20.2%)	4 (4.0%)
NORTON scale – number (%)		
Score improvement	0 (0.0%)	0 (0.0%)
Score worsening	0 (0.0%)	1 (1.0%)
Stable score	14 (14.1%)	12 (12.1%)
Not applicable	85 (85.9%)	86 (86.9%)
CDR scale – number (%)		
Score improvement	1 (1.0%)	7 (7.1%)
Score worsening	14 (14.1%)	11 (11.1%)
Stable score	76 (76.8%)	69 (69.7%)
Not applicable	8 (8.1%)	12 (12.1%)

The analysis of clinical assessment scales also seems to support a more favorable post-implementation course. With regard to nutritional risk, worsening in the MNA score was less frequent after protocol implementation than in the preceding 120 days, while improvements became more common. Functional dependence remained largely stable, which is clinically relevant in such a severely compromised cohort. Similarly, pressure ulcer risk scores were predominantly stable and, in some cases, improved. Meal intake data were also encouraging: across 5,062 recorded dietary days in 50 residents, the average proportion of food consumed remained above 87%, indicating good acceptance of the offered meals.

Finally, clinically relevant adverse events showed a favorable reduction after the implementation of the integrated protocol for dysphagia management. The incidence of pressure ulcers decreased from 24.7% in the pre-implementation phase to 19.2% in the post-implementation phase, corresponding to a 22.3% reduction. Likewise, aspiration pneumonia decreased from 8.2% to 5.1%, corresponding to a 38.7% reduction. Although the observation period was limited to 120 days, these trends are clinically meaningful in a population characterized by very high baseline frailty, severe dependence, and substantial nutritional risk, however, the correlation with the integrated protocol implementation will need to be further investigated in future studies.

### Economic assessment

The cost per dietary day in the pre and post- implementation phase is reported in Table 4. Data shows a cost reduction in the post-implementation phase per dietary day of −1.62 €, corresponding to a 17.5% reduction.

**TABLE 4:** Cost per dietary day in the pre and post implementation phases.

Cost component	Pre implementation phase - mean years 2023/2024	Post implementation phase	Δ
Staff	3.20 €	0.94 €	−2.26 €
Food supply procurement	5.35 €	–	−5.35 €
Outsourced breakfast service	0.51 €	–	−0.51 €
Instant homogenized food	–	6.60 €	+6.60 €
Other indirect costs*	0.20 €	0.10 €	−0.10 €
Total costs	9.26 €	7.64 €	−1,62 €

* “Thickening powders for beverages and pre-packaged gelled drinks” and “ready-to-use foods for dysphagic patients.”

The time dedicated to the preparation of meals is quantified in 11 minutes and 22 seconds considering the pre-implementation phase (considering the time allocated by kitchen staff divided by the total number of dietary days), and in 3 minutes and 21 seconds in the post-implementation phase, considering time needed to prepare the machines, multi-portion dispensing, machines cleaning (considering the mean time per dysphagic subject). Data refers to the observations conducted in two nursing homes and their generalizability should, therefore, be considered low.

### Organizational assessment

The results of the organizational assessment are reported in Table 5, considering elements of value and potential critical issues emerged for each pre identified theme. The main organizational elements of value seems to be related to shorter meal preparation time, standardized consistency ensuring greater safety, improved control of caloric intake, greater uniformity in both dosage and density, improved staff confidence. Potential critical aspects could be related to the need of storage space with monitored environmental humidity levels, machines maintenance and cleaning activity reported as burdensome in some nursing homes.

**TABLE 5:** Organizational assessment results.

Themes	Elements of value	Potential critical issues
Need for additional space	The size of the machines requires limited space in the kitchen and, for breakfast service, within the wards. In all the participating nursing homes, such space was already available.	None.
Adaptation of existing spaces	In most cases, no modification of the kitchen or ward spaces was required in order to use the machines.	In the absence of adequate space, adaptation of the electrical and plumbing systems may be necessary.
	–	Considering product ordering times, additional storage space may be required to ensure supply for periods of 30–40 days.
	–	Environmental humidity levels in the storage area may need to be monitored. If products are not consumed within a few days and humidity is present, there is a risk that the machines may not dispense the product correctly.
Impact on work processes/timing and kitchen staff	Meal preparation times were generally shorter compared with the previous preparation method.	The disassembly, cleaning, and reassembly of the machines were reported as time-consuming by all facilities and more demanding than the previous management approach.
	An advantage in meal service compared with the previous approach was reported, as operators no longer needed to manually adjust product density. The consistency is standardized, ensuring greater food safety.	In some cases, product dispensing failures due to clogged outlets and variations in product density were reported, requiring calibration by the supplying company.
	–	The new dysphagia management approach may require a revision of kitchen work shifts due to the need to prepare meals at the time of lunch and dinner service.
Assistance during meal administration	In some facilities, a shorter time required for breakfast service was observed (estimated reduction of approximately 15 min).	None.
	Staff in some facilities reported feeling more confident thanks to standardized consistency and improved control of caloric intake through standardized portions.	–
Staff training	The training provided by the supplier company was unanimously considered effective. The initial on-site support and subsequent technical assistance were also appreciated.	None.
Quality monitoring	No relevant issues were reported regarding this evaluation aspect, and no changes to current monitoring practices were required.	None.
Acquisition and management costs of the new technology	The contractual conditions generally allowed the cost per dietary day to remain comparable to the previous system.	Some facilities reported the need to reduce the time between product ordering and delivery.
	No significant impact on electricity consumption was reported.	The billing system based on the number of product dispensations is considered complex for operators to record.
Impact on other technologies	From an operational perspective, no reduction in the use of other technologies was observed, as not all residents were managed using the new approach. If applied to all dysphagic residents, a reduced or absent use of blenders and homogenizers is expected.	None.
Economic impact	Some facilities reported that the cost per dietary day was comparable to previous costs.	None.
Management issues and opportunities	In some facilities, the new management approach reduced the use of nutritional supplements.	The need for repeated adjustments of machine parameters by the supplier company was reported as a critical issue, as machines may dispense products with inappropriate consistency until the intervention is performed, requiring operator intervention and reducing the advantage of faster preparation.
	The elimination of subjectivity in preparation and greater uniformity in both dosage and density were positively evaluated by all operators.	Cleaning operations for the machines were considered burdensome by staff.
Need for additional space	The size of the machines requires limited space in the kitchen and, for breakfast service, within the wards. In all the participating nursing homes, such space was already available.	None.
	Meals are prepared and served to residents without additional waiting time.	The impossibility of reheating meals later or providing alternative meals in case of refusal was reported as a limitation.
	For breakfast service, in facilities where breakfast was previously prepared in the kitchen, staff appreciated the possibility of dispensing beverages on demand without the need for reheating.	–
Acceptance by residents	Apart from initial skepticism from some relatives and refusal of certain preparations by some residents, no major critical issues were reported.	None.
Acceptance by staff	Kitchen staff recognized the simplicity of meal preparation.	The main issue reported by kitchen staff relates to daily machine maintenance, including disassembly, washing, and reassembly.
	Care staff generally acknowledged the advantage of serving a product less subject to operator-related variability in terms of consistency, macronutrient and caloric content, and portion uniformity.	Daily maintenance of the breakfast machines was also considered an issue by care staff due to the increase in non-core activities.

## Discussion

The present study should be interpreted against the background of a highly complex clinical population. At baseline, residents showed almost universal nutritional vulnerability, profound functional dependence, advanced cognitive impairment, and high pressure ulcer risk. The coexistence of these factors is important because dysphagia in nursing home residents rarely acts in isolation. Rather, it interacts with frailty, dependency, inflammation, and poor oral intake, thereby generating a self-reinforcing cycle of malnutrition, reduced tissue repair, infections, and further clinical decline. In this context, even partial stabilization of nutritional status may be clinically relevant.

A first major finding of this study is that the integrated protocol appears to have modified the trajectory of deterioration rather than producing a dramatic reversal. Weight and BMI continued to decline on average during the post-implementation period, but the magnitude of reduction was smaller than in the preceding 120 days. In a cohort with severe baseline impairment, this attenuation should not be considered trivial. Preventing or slowing further nutritional decline is itself a clinically meaningful objective in long-term care, particularly when residents are at very high risk of pressure ulcers, aspiration events, and functional worsening.

Bioimpedance findings should be interpreted with caution, but they remain clinically relevant when considered together with the biochemical profile. The increase in phase angle, the rise in intracellular water, the reduction in extracellular water, and the improvement in the ECM/BCM ratio are compatible with a more favorable cellular and hydration pattern. However, as correctly noted by the reviewer, BIA-derived variables may be influenced by hydration shifts, edema reduction, measurement variability, and intercurrent illness, and they should not be interpreted as definitive evidence of muscle gain or structural body-composition recovery, particularly because skeletal muscle mass percentage did not increase. For this reason, the revised interpretation emphasizes a coherent trend toward stabilization and improved body-composition quality rather than a conclusive anabolic effect.

Importantly, despite the stable levels observed, the BIA interpretation was not based on bioimpedance parameters alone. The concurrent improvement in transferrin and total protein, together with the modest but statistically significant increase in albumin, provides an independent biochemical signal compatible with improved protein nutritional status and a more favorable protein-synthesis pattern. These biochemical changes support cautious optimism regarding the BIA findings, because they point in the same direction as the phase angle and ECM/BCM changes. At the same time, they do not eliminate the possibility that fluid redistribution, inflammation, hydration status, renal function, and liver synthetic capacity may have influenced the results.

The observed reduction in aspiration pneumonia and pressure ulcers is clinically consistent with the nutritional and organizational logic of the intervention. A protocol based on safer and more standardized textures, nutritionally controlled meals, intake monitoring, and staff training may plausibly reduce aspiration risk while improving energy-protein exposure and hydration, which in turn may support skin integrity and reduce tissue vulnerability. This interpretation is coherent with the broader literature showing that dysphagia management in nursing homes is most effective when it goes beyond bolus modification alone and includes multidimensional care processes. It is also consistent with evidence showing that non-standardized texture classification can expose residents to harder-than-intended textures and that standardized dysphagia nutrition frameworks may improve safety and intake (11, 14). It should be noted that the 120-day observation period considered in the analysis is relatively short for detecting substantial nutritional recovery, functional changes, or variations in the incidence of clinical events. However, given the high level of frailty of the study population, a longer follow-up period was considered less appropriate, as nursing home residents meeting the inclusion criteria would be at considerable risk of not surviving.

The study also aligns with the emerging view that meal quality and acceptability are central to dysphagia care. Sukkar et al. previously showed that instant homogenized meals were associated with higher consumption and satisfaction than traditional pureed foods, while Pedrolli highlighted the need for clearer dysphagia-specific nutritional standards and standardized texture governance. In the present study, the high average meal consumption observed in food diaries and the generally favorable organizational feedback from staff reinforce the idea that a dysphagia protocol should be judged not only on safety outcomes, but also on its ability to support actual intake, standardization, and workflow feasibility (17, 26).

From an organizational and economic perspective, the findings are promising but should be interpreted pragmatically. The protocol appears to reduce preparation time, improve confidence in consistency control, and support better monitoring of caloric intake. Nevertheless, some of the potential economic savings, particularly those related to staff time, remain partly theoretical in the short to medium term because food-service personnel are often a fixed cost for facilities. In addition, the organizational balance may differ substantially across nursing homes depending on kitchen workflows, procurement arrangements, storage capacity, and the burden of machine maintenance. For this reason, the main strength of the protocol may lie less in universal cost reduction and more in process standardization, reproducibility, and risk control. As previously reported, pre-implementation costs could not always be specifically isolated for residents with dysphagia. Therefore, results should be considered with caution.

Important limitations must be emphasized. First, the uncontrolled single-group before-and-after design prevents causal attribution to the integrated protocol, as temporal trends, changes in staffing, seasonal effects, regression to the mean, survivor bias, and unmeasured changes in clinical management may have contributed to the observed differences. Second, missing data were present for several laboratory and body-composition variables, reflecting the retrospective use of routine clinical records; therefore, estimates for these parameters should be considered less robust than outcomes with more complete data. Third, the absence of covariate adjustment means that age, sex, dementia severity, baseline nutritional status, comorbidity burden, facility-level variability, and dysphagia severity could not be accounted for. Fourth, the qualitative organizational component was exploratory and pragmatic: although interviews were useful to identify recurrent implementation issues, no formal coding framework, saturation assessment, or theory-driven qualitative methodology was applied. These limitations reinforce the need for larger prospective controlled studies before drawing definitive conclusions regarding efficacy or cost-effectiveness.

Overall, the present findings suggest that integrated dysphagia management based on standardized instant homogenized meals, structured nutritional monitoring, and continuous staff training may contribute to short-term clinical stabilization in highly frail nursing home residents. The most robust signals concern attenuation of nutritional decline, good meal acceptance, selected biochemical improvements in protein-related markers, and BIA-derived changes that are directionally consistent with better body-composition quality, while remaining potentially influenced by fluid shifts and measurement variability. These findings should be confirmed in larger controlled studies with longer follow-up, but they support the view that dysphagia management in nursing homes should be approached as a combined clinical, nutritional, and organizational intervention rather than as a simple texture modification strategy.

## Data Availability

The original contributions presented in this study are included in this article/supplementary material, further inquiries can be directed to the corresponding author.
